# Correction: Effect of fatty acids on the accelerated sulfur vulcanization of rubber by active zinc/carboxylate complexes

**DOI:** 10.1039/d5ra90128f

**Published:** 2025-11-07

**Authors:** Preeyanuch Junkong, Rie Morimoto, Kosuke Miyaji, Atitaya Tohsan, Yuta Sakaki, Yuko Ikeda

**Affiliations:** a Center for Rubber Science and Technology, Kyoto Institute of Technology Matsugasaki, Sakyo Kyoto 606-8585 Japan yuko@kit.ac.jp; b Faculty of Science, Mahidol University Ratchathewi Bangkok 10400 Thailand; c Graduate School of Science and Technology, Kyoto Institute of Technology Matsugasaki, Sakyo Kyoto 606-8585 Japan; d Faculty of Engineering, King Mongkut's University of Technology North Bangkok 1518 Pracharat 1 Rd, Wongsawang, Bangsue Bangkok 10800 Thailand; e Faculty of Molecular Chemistry and Engineering, Kyoto Institute of Technology Matsugasaki, Sakyo Kyoto 606-8585 Japan

## Abstract

Correction for ‘Effect of fatty acids on the accelerated sulfur vulcanization of rubber by active zinc/carboxylate complexes’ by Preeyanuch Junkong *et al.*, *RSC Adv.*, 2020, **10**, 4772–4785, https://doi.org/10.1039/C9RA10358A.

The ORCiD previously listed for Yuko Ikeda was incorrect. The correct ORCiD is: 0000-0003-2809-8911.

The Royal Society of Chemistry apologises for these errors and any consequent inconvenience to authors and readers.

